# Supplementary figures and images for: Genetic and compound screens uncover factors modulating cancer cell response to indisulam
Source: Life Sci Alliance. 2022 May 9;5(9):e202101348. doi: 10.26508/lsa.202101348 (PMC9095732; doi:10.26508/lsa.202101348)

GAPDH  
Used in figure 1B

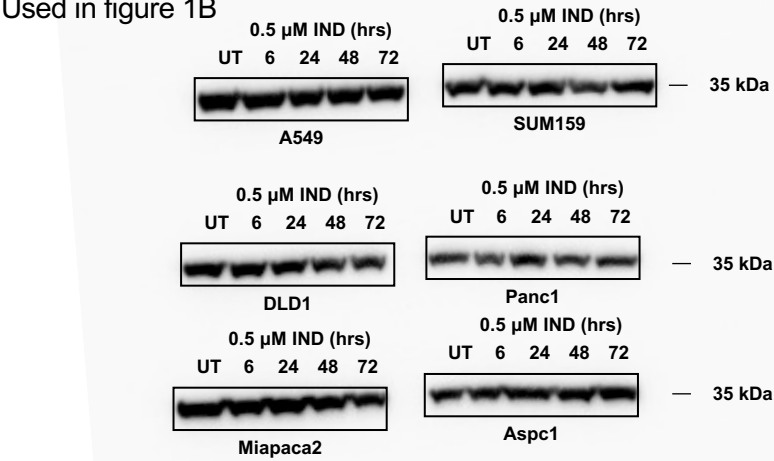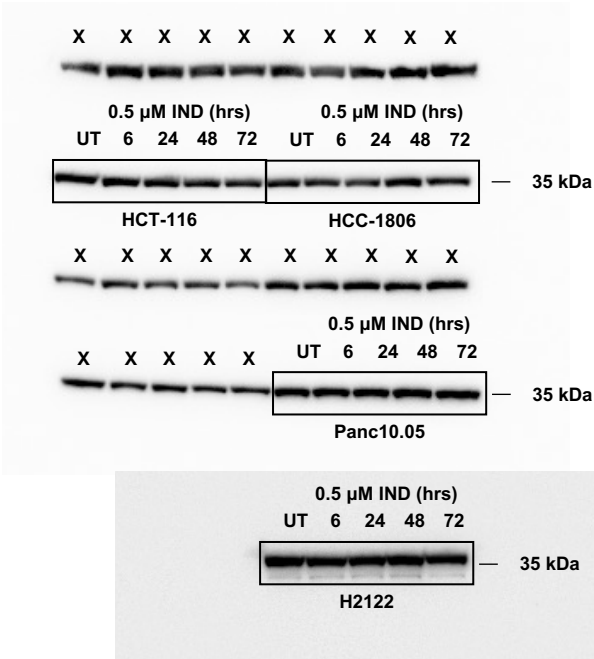

RBM39  
Used in figure 1B

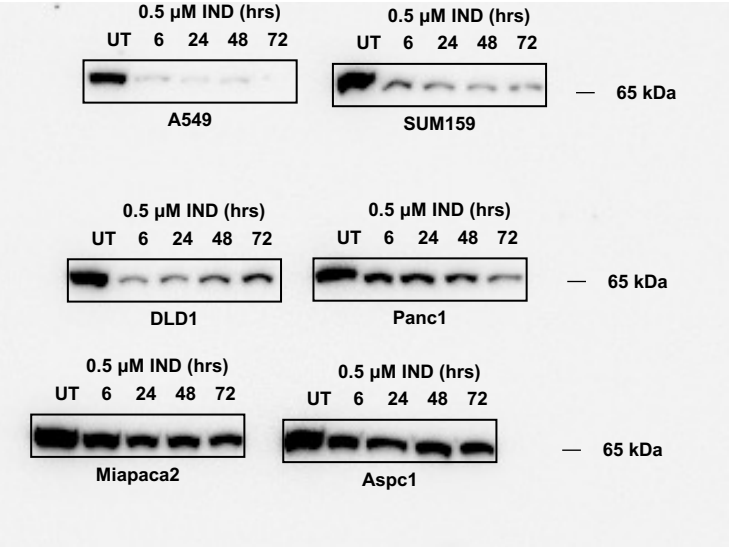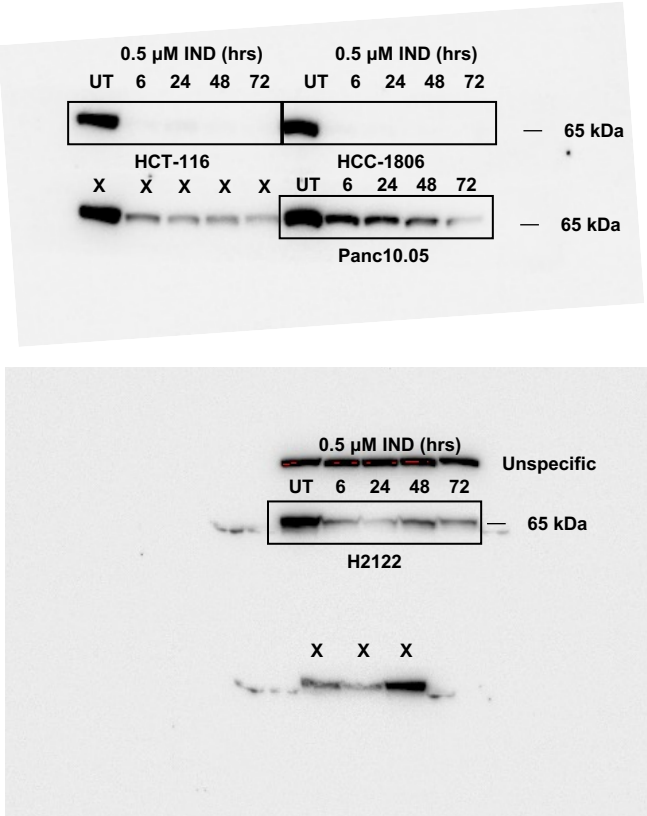

GAPDH  
Used in figure1D

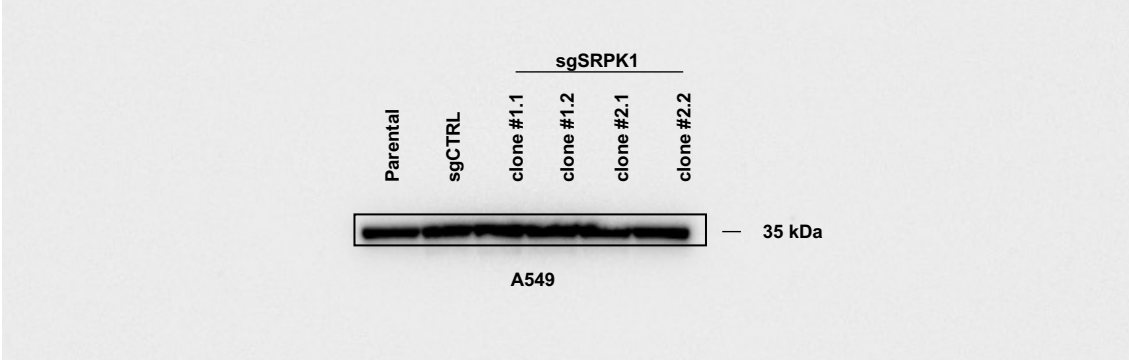

SRPK1  
Used in figure1D

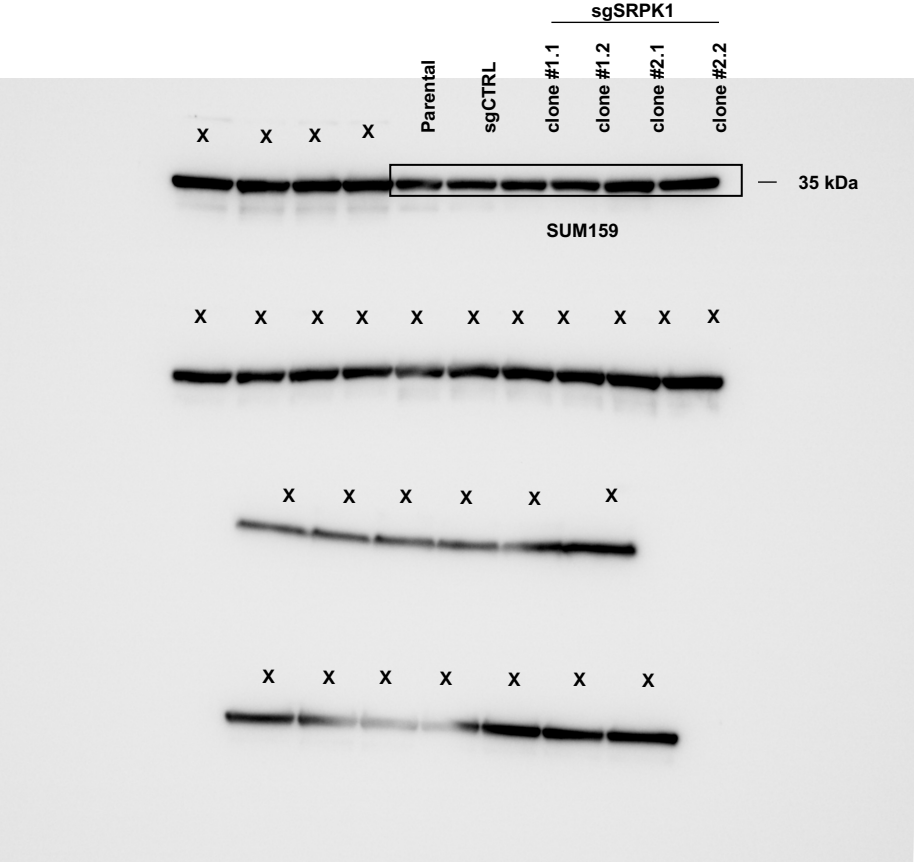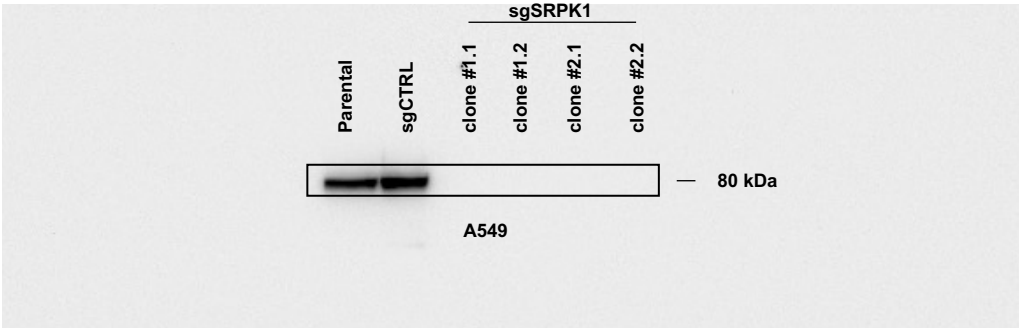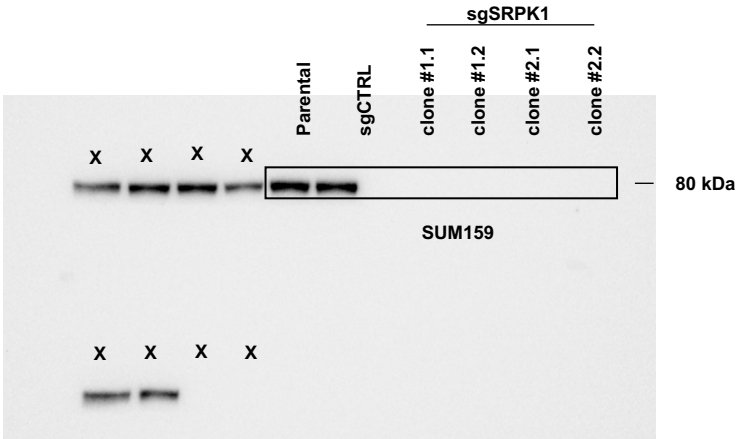

Supplement: Supplementary file 1 [file LSA-2021-01348_SdataF1.1.pdf]

GAPDH  
Used in figure 2D

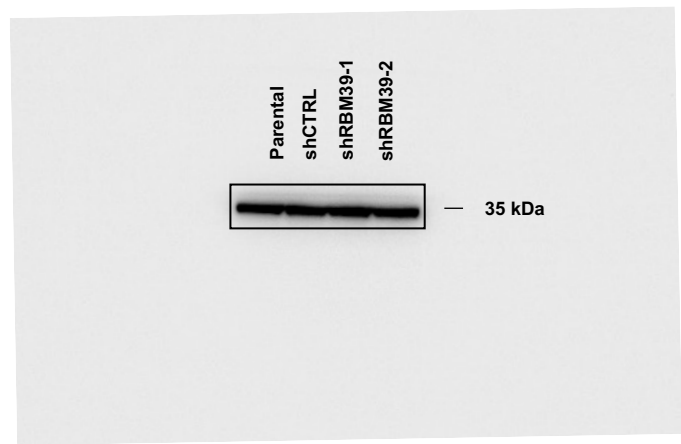

RBM39  
Used in figure 2D

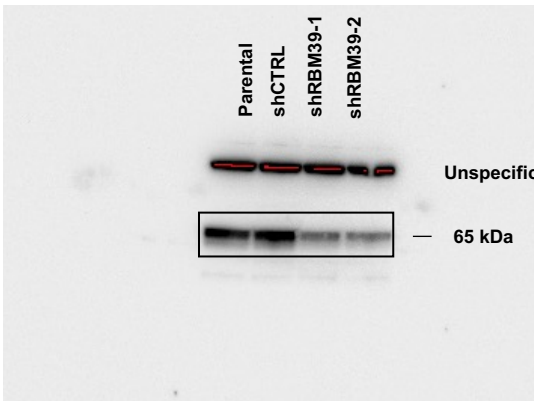

Supplement: Supplementary file 3 [file LSA-2021-01348_SdataF2.1.pdf]

Tubulin  
Used in figure 4C

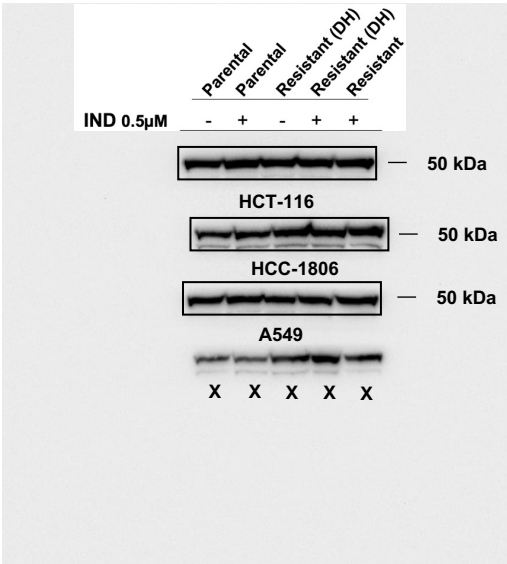

RBM39  
Used in figure 4C

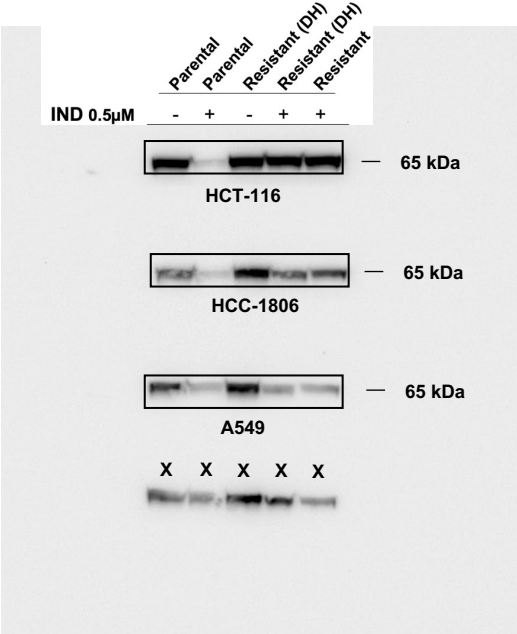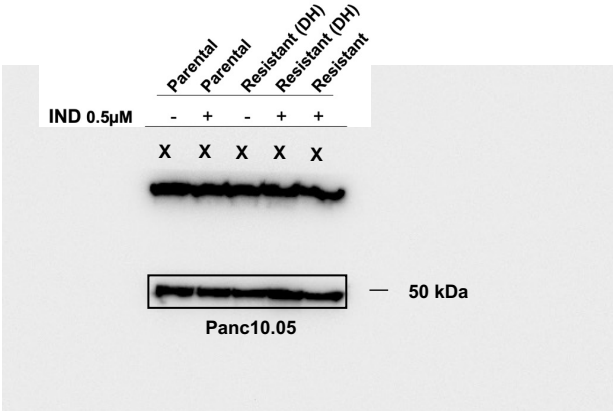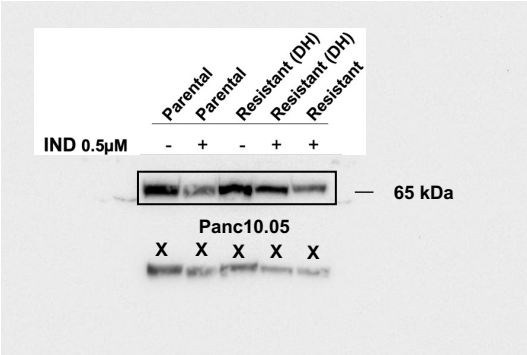

Vinculin  
Used in figure 4H

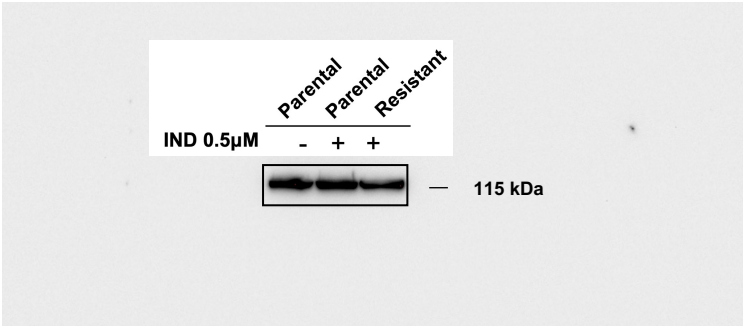

Bcl-xL  
Used in figure 4H

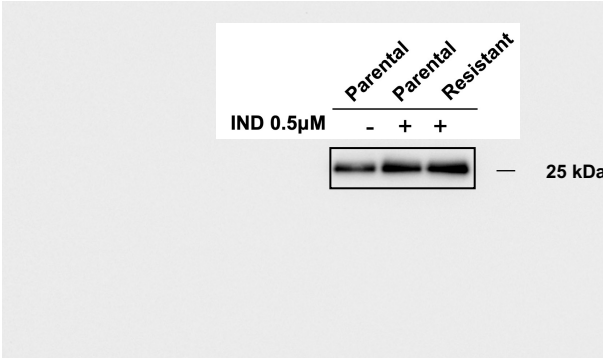

Supplement: Supplementary file 7 [file LSA-2021-01348_SdataF4.1.pdf]
