# Supplementary material for: Genetic and compound screens uncover factors modulating cancer cell response to indisulam
Source: Life Sci Alliance. 2022 May 9;5(9):e202101348. doi: 10.26508/lsa.202101348 (PMC9095732; doi:10.26508/lsa.202101348)

GAPDH
Used in figure 3D,F

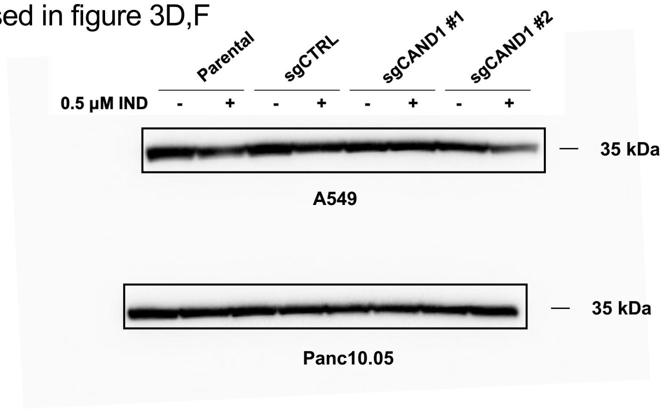

RBM39
Used in figure 3D,F

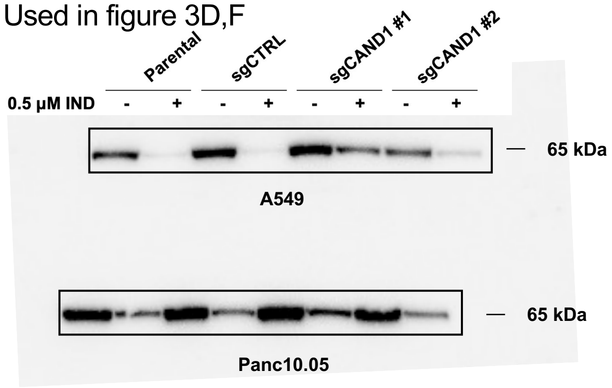

CAND1
Used in figure 3D,F

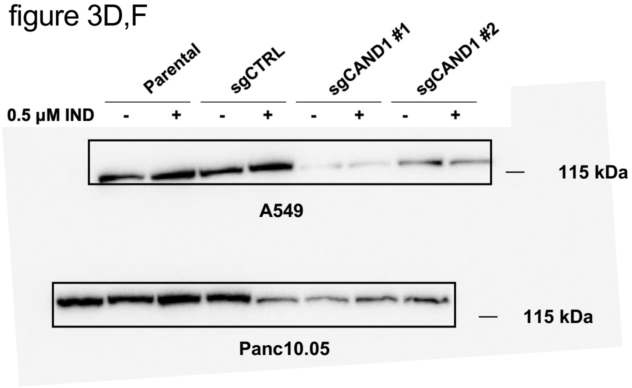

GAPDH
Used in figure 3H

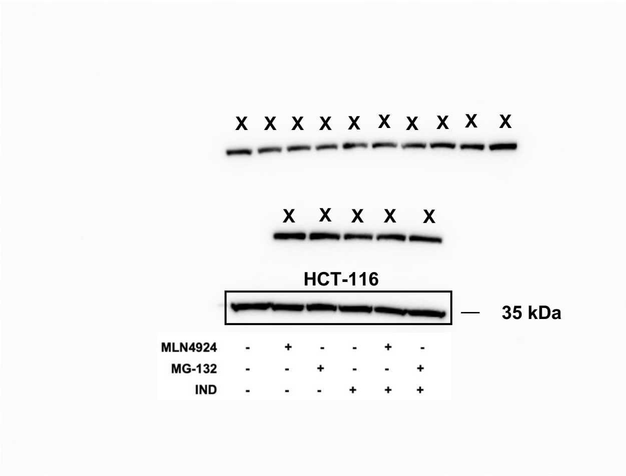

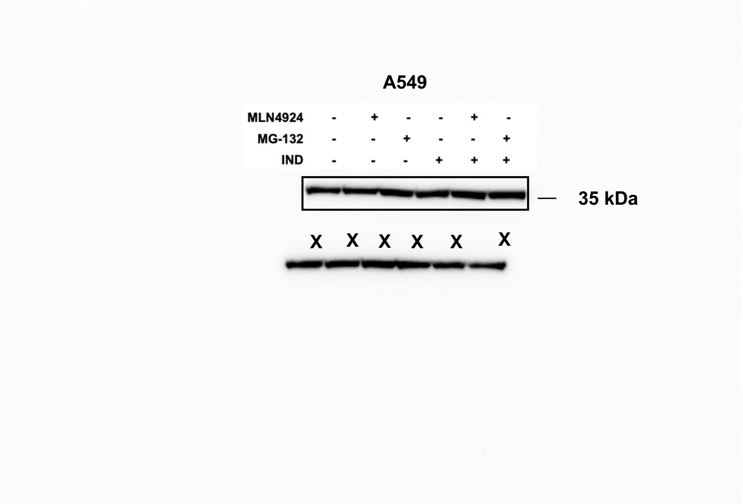

CUL4A
Used in figure 3H

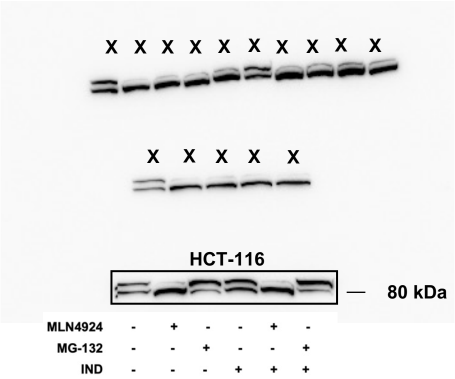

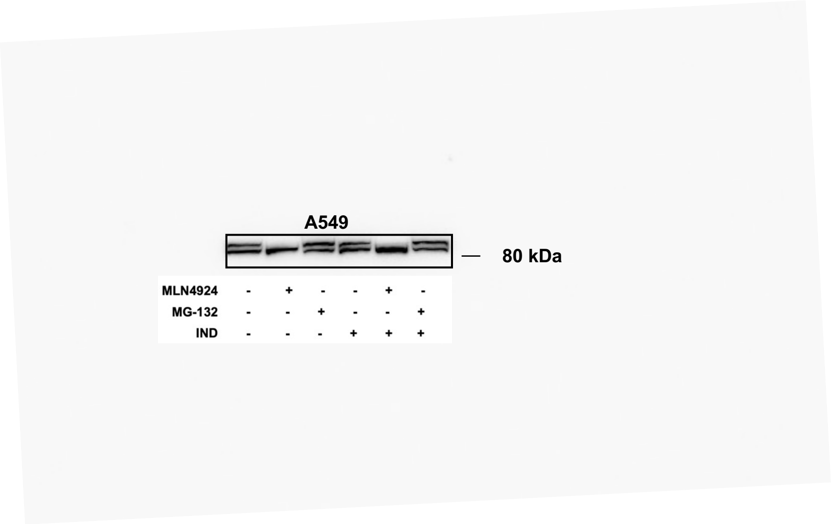

RBM39
Used in figure 3H

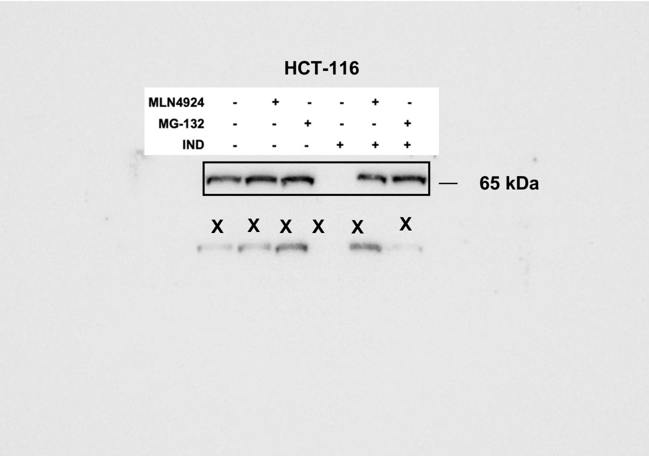

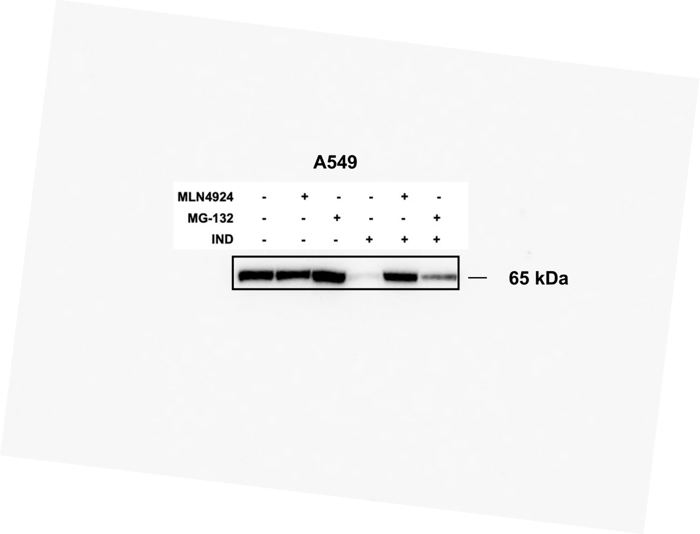

GAPDH
Used in figure 3I

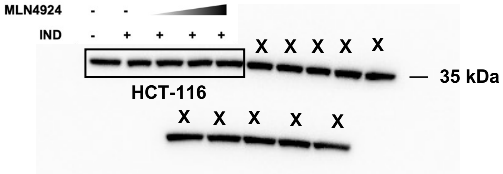

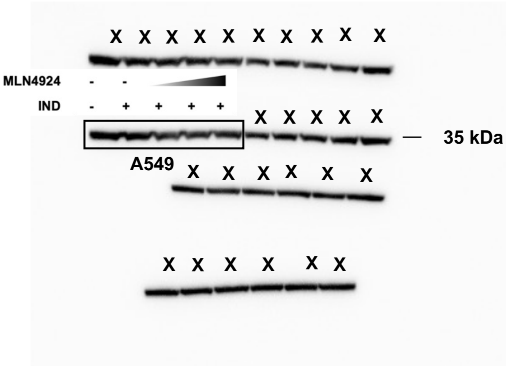

CUL4A
Used in figure 3I

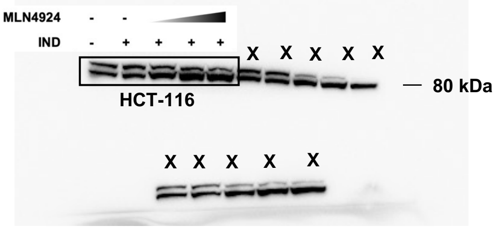

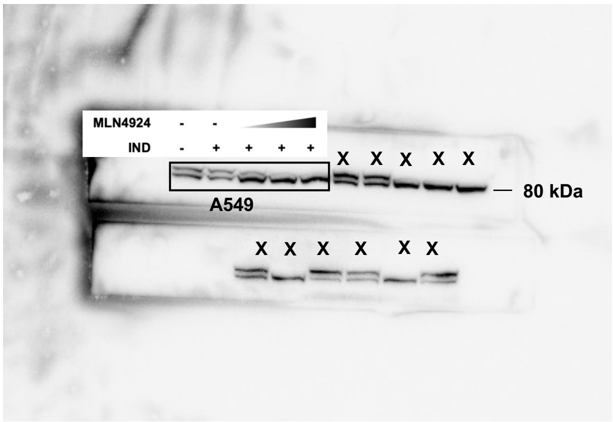

RBM39
Used in figure 3I

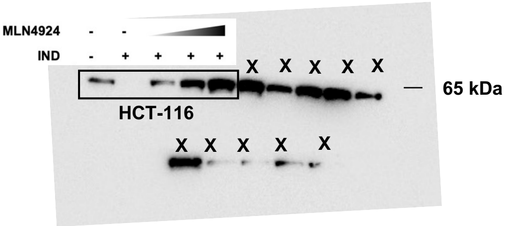

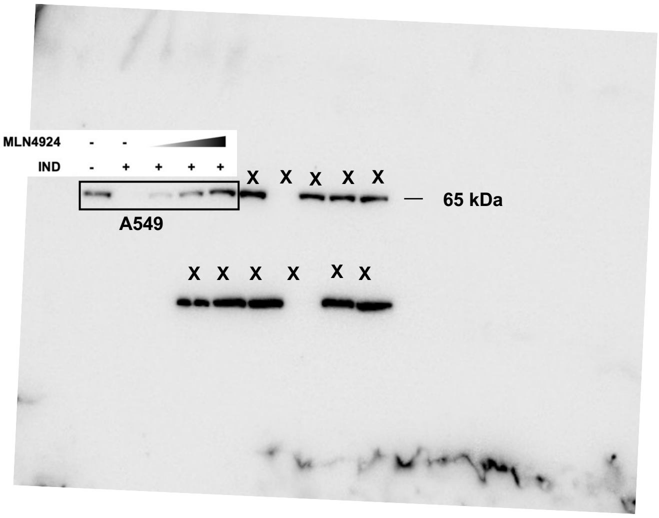

Supplement: Supplementary file 5 [file LSA-2021-01348_SdataF3.1.pdf]
